# *Metarhizium anisopliae* Pathogenesis of Mosquito Larvae: A Verdict of Accidental Death

**DOI:** 10.1371/journal.pone.0081686

**Published:** 2013-12-13

**Authors:** Tariq M. Butt, Bethany P. J. Greenfield, Carolyn Greig, Thierry G. G. Maffeis, James W. D. Taylor, Justyna Piasecka, Ed Dudley, Ahmed Abdulla, Ivan M. Dubovskiy, Inmaculada Garrido-Jurado, Enrique Quesada-Moraga, Mark W. Penny, Daniel C. Eastwood

**Affiliations:** 1 College of Science, Swansea University, Swansea, United Kingdom; 2 College of Engineering, Swansea University, Swansea, United Kingdom; 3 College of Medicine, Swansea University, Swansea, United Kingdom; 4 Institute of Systematics and Ecology of Animals, Siberian Branch of Russian Academy of Sciences, Novosibirsk, Russia; 5 Department of Agricultural and Forestry Sciences, University of Cordoba, Cordoba, Spain; Universidad Autónoma del estado de Morelos, Mexico

## Abstract

*Metarhizium anisopliae*, a fungal pathogen of terrestrial arthropods, kills the aquatic larvae of *Aedes aegypti*, the vector of dengue and yellow fever. The fungus kills without adhering to the host cuticle. Ingested conidia also fail to germinate and are expelled in fecal pellets. This study investigates the mechanism by which this fungus adapted to terrestrial hosts kills aquatic mosquito larvae. Genes associated with the *M. anisopliae* early pathogenic response (proteinases *Pr1* and *Pr2*, and adhesins, *Mad1* and *Mad2*) are upregulated in the presence of larvae, but the established infection process observed in terrestrial hosts does not progress and insecticidal destruxins were not detected. Protease inhibitors reduce larval mortality indicating the importance of proteases in the host interaction. The *Ae. aegypti* immune response to *M. anisopliae* appears limited, whilst the oxidative stress response gene encoding for thiol peroxidase is upregulated. *Cecropin* and *Hsp70* genes are downregulated as larval death occurs, and insect mortality appears to be linked to autolysis through caspase activity regulated by *Hsp70* and inhibited, in infected larvae, by protease inhibitors. Evidence is presented that a traditional host-pathogen response does not occur as the species have not evolved to interact. *M. anisopliae* retains pre-formed pathogenic determinants which mediate host mortality, but unlike true aquatic fungal pathogens, does not recognise and colonise the larval host.

## Introduction

Mosquitoes vector a wide range of diseases (e.g. dengue, yellow fever and malaria) which can have a devastating impact on human health. Almost half the world's population is at risk to mosquito-transmitted diseases and the range has expanded due to climate change and increased trade [1]. Many chemical pesticides have been withdrawn due to the risks they pose to humans and the environment, and development of resistance in pest populations. Recent studies show that *Metarhizium anisopliae,* a soil borne fungal pathogen of terrestrial insects, offers an environmentally friendly alternative to chemicals for the control of mosquitoes. *M. anisopliae* will kill adult and larval stages of *Aedes*, *Anopheles* and *Culex* mosquitoes [2], [3] yet the mechanism of how this terrestrial pathogen kills the aquatic larval stage is unclear. Strains of *M. anisopliae* have been developed to control a wide range of terrestrial arthropods including pests of agro-forests crops and vectors of human and animal diseases [4], [5]. Infection of terrestrial arthropod hosts by *M. anisopliae,* like that of most other entomopathogenic fungi, follows a consistent pattern. Firstly, spores adhere to the surface of the host cuticle followed by germination and differentiation of an appressorium from which a narrow penetration peg is produced which penetrates the cuticle using a combination of enzymes and mechanical force [6], [7]. Following colonization of the hemocoel, the fungus erupts through the intersegmental membranes and differentiates conidiophores and conidia. The key pathogenicity determinants of *M. anisopliae* include cuticle degrading enzymes like *Pr1* (subtilisin protease) and toxic cyclic peptides like destruxins [8]. Fungal virulence appears to be correlated with *Pr1* and destruxin production; with hyper producers being more virulent [8]. Equally important are the adhesins, *Mad1* and *Mad2*, which play an important role in adhesion of *M. anisopliae* to the insect cuticle [9]. Disruption of the *Mad1* gene reduces virulence by reducing adhesion of conidia to the host surface [10]. It is assumed that the pattern of infection outlined above occurs in aquatic mosquito larvae [2].This paper demonstrates for the first time that *M. anisopliae* kills the mosquito larvae *via* a mechanism which does not entail the traditional infection processes.

Based on the limited number of observational studies conducted on *M. anisopliae* infection of mosquito larvae, possible routes of invasion have been reported including entry *via* penetration of the cuticle, the respiratory siphon or alimentary canal, however, the precise mechanism remains elusive. Lacey *et al*., [11] noted that when larvae of *Culex quinquefasciatus* broke the water tension with their perispiracular valves for air intake, floating conidia of *M. anisopliae* adhered to the inside surface of the valves, germinated and invaded the siphon tip tissue, then extended into and blocked the trachea resulting in suffocation and death. Lacey *et al*., [11] also noted that conidia suspended in the water were ingested and occluded the larval gut, initiating mortality within 6 to 24 hr after ingestion. In contrast, Riba *et al*., [12] reported that *M. anisopliae* conidia killed *Ae. aegypti* within 1.1 days before intra-hemocoelic invasion. Some workers suggest that death is due to colonization of the hemocoel by the fungus [2], [12], others suggest it is due to toxins released by ingested conidia without colonisation of the hemocoel [11], [13].

## Materials and Methods

### Fungal strains and production

*Metarhizium anisopliae* isolate ARSEF 4556, identified as highly pathogenic to mosquitoes and midges [4], was maintained on Sabouraud dextrose agar (SDA) or broken Basmati rice [14]. Conidia used in assays had over 95% viability. A green fluorescence protein (GFP) transformed strain of *Metarhizium brunneum* EAMa 01/58 Su was maintained on SDA.

### Mosquito source and maintenance

*Aedes aegypti* (strain AeAe) eggs, obtained from the London School of Hygiene and Tropical Medicine, were hatched in distilled water and the larvae fed on Tetramin® fish food, at room temperature (22°C±2°C).

### Inoculation of *Aedes* larvae with *Metarhizium* conidia

Assays were performed using 24 well plates (Nunc, Roskilde, Denmark) with one larva per well. *M. anisopliae* ARSEF 4556 was assayed at 10^7^ conidia ml^−1^ against L_3−4_ larvae. Additional assays were done using heat killed conidia to determine the role of extracellular enzymes in pathogenesis. Extracellular enzymes were denatured by wrapping the conidia in aluminium foil and autoclaving for 15 min at 121°C. Conidial viability was assessed using the plate count technique [15]. Control larvae were exposed to either 1ml 0.03% Aq Tween 80 or distilled water. Larval mortality was recorded daily up to 7 days. All assays were performed at room temperature with a 16L:8D photoperiod. There were 24 larvae per assay which was repeated three times. This format was used in subsequent assays to study host-pathogen interactions, in particular, insect defense responses and regulation of *M. anisopliae* pathogenicity determinants.

Larvae (n = 20) were inoculated with conidia of ARSEF 4556 as outlined above and examined at 0, 24, 48 and 72 hr post inoculation (pi). Healthy and infected larvae were examined by light microscopy (LM) to determine if there were preferential sites for spore adhesion and to monitor passage of the fungal conidia through the gut. Larvae (n = 20) were also examined by cryo scanning electron microscopy (SEM) using a Hitachi S4800 field emission microscope equipped with a Quorum PPT2000 cryogenic stage and preparation chamber. Full details on the cryo-SEM are provided in Text S1 in File S1. Additional studies were done using a GFP-transformed strain of *M. brunneum.* The surface and gut contents of infected *Ae. aegypti* larvae (n = 10)as well as fecal pellets were examined by fluorescence microscopy (FM) using a Zeiss fluorescence microscope.

### Mass spectrometry analysis of destruxins

Assays were performed using 24-multi-well plates with ten *Ae. aegypti* larvae (L_3–4_) per well containing 1 ml aqueous suspension of 1×10^7^ conidia ARSEF 4556 or 1 ml of distilled water (control). After 24 hr incubation, the larvae were removed and prepared for destruxin extraction and analysis by mass spectroscopy as described by Butt *et al*., [16]. The effect of destruxins on *Ae. aegypti* larvae was also tested by introducing larvae to 1 ml of distilled water spiked with 1nmole of destruxin A (Sigma-Aldrich) and determining larval survival. Briefly, destruxin extracts were analyzed by nano-reverse phase liquid chromatography (Ultimate Pump, LC-Packing, Dionex, The Netherlands) using an electrospray ion trap MS (LCQ Deca XP, ThermoElectron, Hemel Hempstead, UK). Matrix Assisted Laser Deionisation Mass Spectroscopy (MALDI MS) was used to confirm the levels of detection below which any destruxins might be exhibited within the larvae themselves. For this purpose, larval extract was re-dissolved in a matrix solution and 1 µl of the resulting mixture was spotted onto the MALDI plate and allowed to dry at room temperature. A 10 mg/mL alpha cyanocinnaminic acid (CHCA) in 50∶50 0.1% trifluoroacetic acid (TFA): acetonitrile (ACN) matrix solution was used and a Voyager DE-STR instrument (Applied Biosystems, UK) was utilized in reflectron mode. An acceleration voltage of 20,000V and a grid voltage of 70% was utilized in order to study any peptides present and a 1pmole standard of destruxin A applied to the plate as a control and as a method of determining the level below which any destruxins are present if signal intensity were below the standard. Full details are provided in Text S2 in File S1.

### Enzymology

#### (i) Protease inhibition assays

To determine if the extracellular proteases were responsible for larval mortality. Larvae (n = 24) were exposed to *M. anisopliae* ARSEF 4556 conidia containing either chicken egg white (0.1 mg/ml), EDTA (1 mM) or α2-macroglobulin (1 µg/ml) which were inhibitors specific for *Pr1*, metalloprotease and global (serine, cysteine, metallo-) proteases, respectively. All the inhibitors were purchased from Sigma-Aldrich. Controls consisted of buffer and buffered inhibitor. Mortality was recorded at 0, 12, 24, 36, 48 and 72 hr pi. Assays were also done using heat killed conidia at 10^7^ conidia ml^−1^.

#### (ii) Caspase assays

Activity of caspases 2, 3, 7 and 8 was assayed using luminometric kits in accordance with the manufacturer's guidelines (Promega). Six larvae were examined per treatment with the endpoint luminescence being measured after 1 hr, in four replicate wells for each larvae. Full details are given in Text S3 in File S1.

### Transcript quantification of insect and fungus-derived genes

#### (i) Samples, RNA extraction and cDNA synthesis

Full details of the transcript quantification are given in Text S4 in File S1. Briefly, *Ae. aegypti* larvae (L_3–4_) (n = 3, 10 larvae per replicate) were exposed to *M. anisopliae* ARSEF 4556, controls included larvae not exposed to fungus and a terrestrial insect, *Tenebrio molitor*. Samples were frozen under liquid nitrogen and stored at −80°C until required. All samples were ground with a micropestle and total RNA extractions carried out using the RNeasy Micro kit (Qiagen) following the manufacturer's instructions. RNA concentration and purity was assessed at 260 and 280 nm absorbance using a Nanophotometer (Implen). Total RNA (1 μg) was either RQ1 RNase-free DNAse (Promega) treated and reverse transcribed using the qScript cDNA synthesis kit (Quanta Biosciences), or reverse transcribed using the QuantiTect Reverse Transcription kit (Qiagen) with gDNA elimination reaction, for the experiment to quantify insect-derived transcripts and fungus-derived transcripts, respectively. Relative cDNA quantity was analyzed by PCR using two reference genes for insect or fungal cDNA samples to ensure consistency between values: *Ae. aegypti* ribosomal S7 (accession number: AAEL009496) and ribosomal protein 49/L32 (AAEL003396) and *M. anisopliae* 18S rRNA and elongation factor tEF (Table S1 in File S1).

#### (ii) Quantitative PCR (qPCR)

Transcript levels were determined using the Rotor-Gene 6000 (Corbett Research) or CFX96™ Real-Time PCR detection system (Biorad) for *Ae. aegypti* and *M. anisopliae* gene targets respectively. Primers were designed to amplify key *Ae. aegypti* response genes and *M. anisopliae* pathology-related genes (Table S1 in File S1).

The accompanying software for each qPCR instrument was used to analyze the raw data and carry out quality control for each sample. The cycle threshold (Ct) value was determined for each reaction and normalized to the geometric mean of the appropriate endogenous reference genes. Relative gene expression was calculated using the comparative C_t_ method (2-ΔΔC_t_) following established methodology [17].

### ROS production, lipid peroxidation and antioxidant system activity

Three insects per time point were sample were homogenized in 100 µl of ice-cold phosphate buffered saline (10 mM phosphate buffer, 150 mM NaCl, pH 7.2) containing phenylthiourea (1 mg/ml). The homogenate was centrifuged for 5 min, 10,000 g at 4°C and activities determined as described by Dubovskiy *et al*.[18], in 12 replicates for ROS production and Lipid peroxidation and 9 replicates for Super Oxide Dismutase (SOD), Glutathione-S-transferase (GST) and Catalase activity. Full details are provided in Text S5 in File S1.

### Statistical Analysis

Differences in mosquito larvae survival between live and heat killed conidia and protease inhibited conidia were analysed using Kaplan-Meier survival analysis to plot cumulative survival functions by treatment with pairwise comparison over log-rank test [4]. Biochemical and molecular data sets were analyzed using two-way Analysis of Variance (ANOVA) with Bonferroni's post-test. Prior to analysis gene expression data was logarithm (base 10) transformed, conforming to ANOVA assumption of homogeneity of variance [19]. All statistical analyses were carried out using SPSS v21.0 [20] and GraphPad Prism v5.0 (GraphPad Software, USA).

## Results

### Virulence of *Metarhizium anisopliae* ARSEF 4556 for *Aedes aegypti* larvae

**Figure 1:**
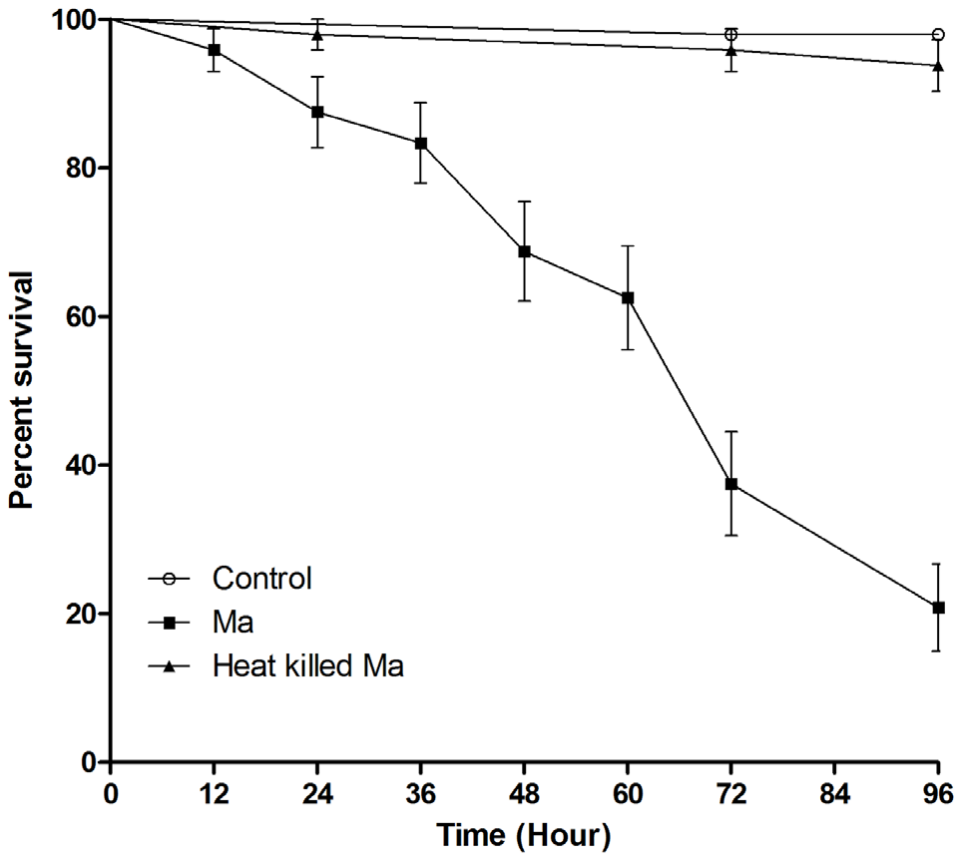
Heat killed treatment increased the survival of *Ae. aegypti* larvae. Late 3^rd^−4^th^ instar *Ae. aegypti* larvae (n = 72) were inoculated with live and heat killed conidia of *M. anisopliae.* Kaplan-Meier method was used to plot cumulative survival curves of larvae after inoculation, log-rank test was used to assess difference in survival between treatments. The curves of non-exposed and heat killed treatment show no statistical difference. Live conidia significantly decreased survival compared to heat killed conidia (p<0.001). Larvae with no fungal treatment were used as a negative control.The aim of this study was to establish the cause of death as this could have profound implications on the formulation and deployment of *Metarhizium* and other terrestrial entomopathogenic fungi to control mosquito larvae. This study, utilising a combination of microscopy, molecular and biochemical methods, shows that *Metarhizium* invasive strategies, evolved for terrestrial hosts including adult mosquitoes, have not evolved for killing aquatic insect hosts with resultant mortality in mosquito larvae being multi-factorial.

*Ae. aegypti* larvae were highly susceptible to *M. anisopliae* 4556 with the earliest mortalities being observed 24 hr pi and percentage mortality reaching 60%−90% between 72 and 96 hr pi (Fig. 1). Heat killed conidia were used to determine whether conidia caused blockage within the larvae or were required to actively bring about mortality. Mortality was significantly higher in larvae exposed to live conidia than those exposed to heat killed conidia (*p*<0.001) and there was no significant difference in mortality between the heat killed conidia (*p*<0.153) and the untreated control (Fig. 1).

### *Metarhizium* conidia fail to infect *Aedes* larvae

**Figure 2:**
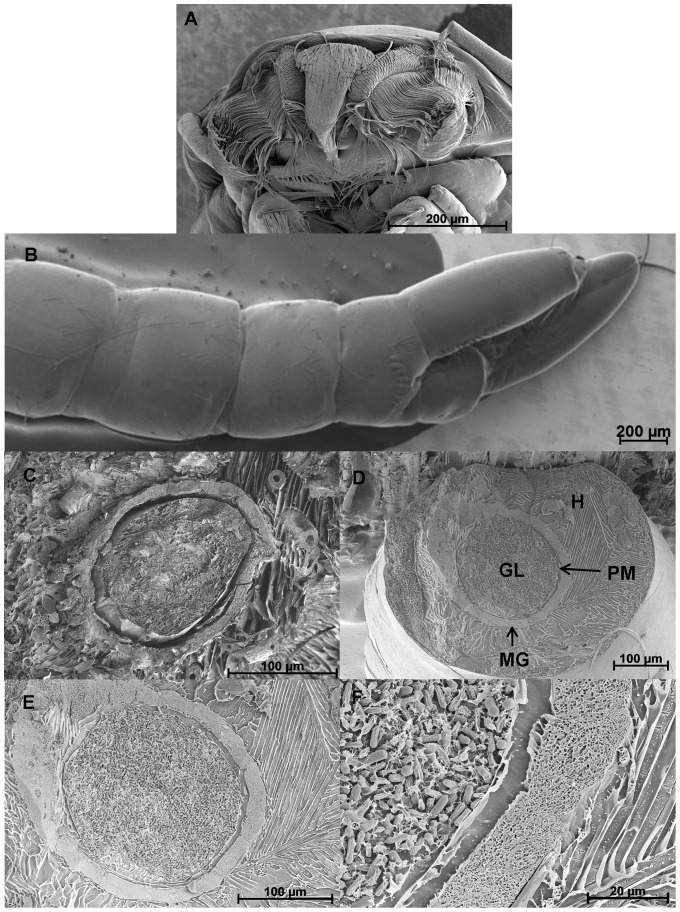
*M. anisopliae* do not attach to or invade mosquito hemocoel. Larvae inoculated with 1×10^7^ conidia ml^−1^ *M. anisopliae*, 48 hr post-inoculation, were subjected to Cryo-SEM to establish areas of attachment and penetration of the fungus. No conidia were observed attached to the surface of the head (A), abdomen and siphon (B). Cross section of control larva (C) and infected larva (D) showing the gut lumen (GL), midgut epithelium (MG), and peritrophic matrix (PM). Conidia of *M. anisopliae* were restricted to and appeared to occlude the gut lumen. (D−E). Both swollen and collapsed conidia were observed in the gut lumen with no evidence of conidia penetrating the gut wall (F) and invading the hemocoel (H).Light, fluorescence and scanning electron microscopy

**Figure 3:**
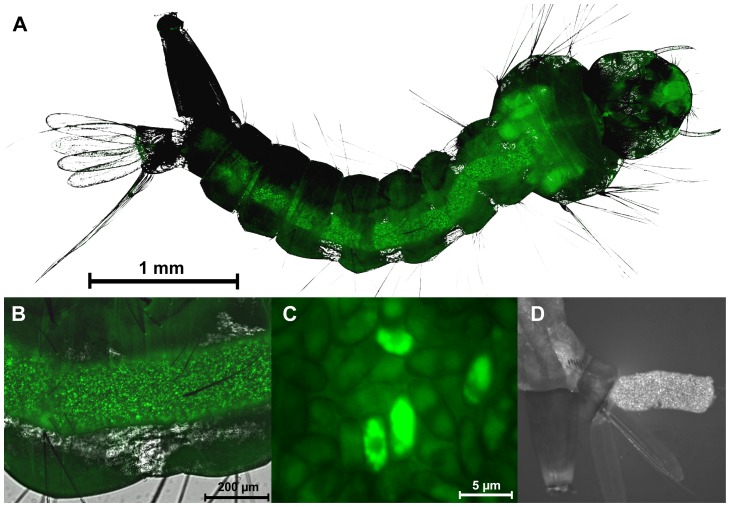
*Metarhizium* conidia expressing GFP in the gut and fecal pellets demonstrating activity and viability. Larvae inoculated with conidia of a GFP transformed strain of *Metarhizium brunneum* were examined 48 hr pi to assess viability and activity of the conidia. (A) Conidia occlude most of the gut lumen, some autofluorescence is seen in the head and thorax region. (B) Numerous conidia are active and expressing the GFP within the gut lumen. (C) High magnification of conidia expressing GFP, the non-fluorescing conidia may be inactive or dead, (D) Fecal pellet being expelled from an infected larva showing many active conidia.

Microscopy studies clearly showed little or no attachment of conidia to the surface of the mosquito body (Figs. 2A and B) with conidia being concentrated in the gut lumen (Figs. 2D-F). In some larvae, the conidia occluded the gut lumen while in others gaps were observed between the conidial clumps. None of the conidia in the gut had produced a germ tube and whereas some conidia were hydrated and swollen others appeared collapsed (Fig. 2F). Conidia had a prominent hydrophobin rodlet layer with little evidence of mucilage secretion. FM showed that conidia in the gut and those present in fecal pellets expressed the GFP and were clearly viable and active (Fig. 3). Most non-fluorescing GFP conidia were probably quiescent.

SEM examination of cross sections of abdomen showed no obvious differences in the appearance of the gut epithelial cells, peritrophic matrix and other structures of treated and untreated (control) larvae (Figs. 2C and D–F). There was no visible evidence of damage to the internal organs. Compact fecal pellets were produced by control and infected larvae (Fig. 3) suggesting the peritrophic matrix, peristalsis and other gut functions were intact at least until the time of death. The fungus never crossed the gut; it did not colonize the hemocoel as it would terrestrial arthropod hosts.

### Destruxins are not the cause of larval death

**Figure 4:**
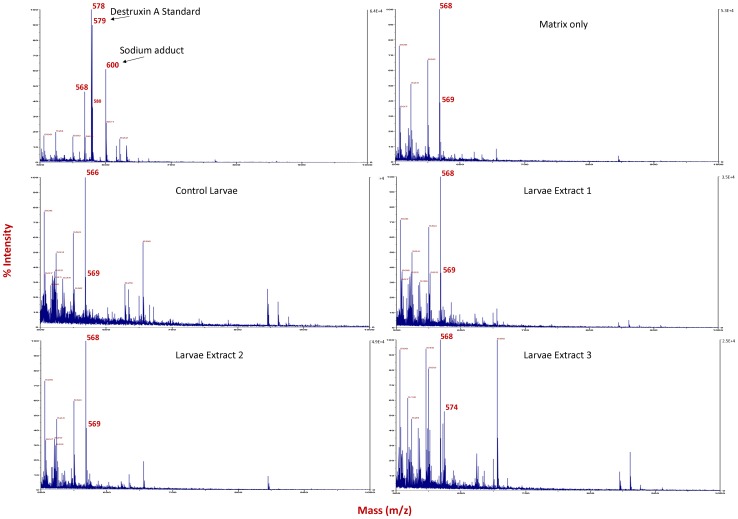
Destruxins not detected in larval extracts. Larval extracts were analysed by MALDI ToF alongside a destruxin A standard. No destruxin signals were detected with both larval extract and control larvae containing only those ions formed due to the MALDI matrix itself.

Destruxins, common virulence determinants of *M. anisopliae,* were not detected by mass spectroscopy in *Ae. aegypti* larvae that had ingested conidia of *M. anisopliae.* The LCMS analysis was utilized to profile for any destruxin signals present using constant neutral loss signals established in our laboratories that highlight low levels of destruxins [16]. No signals were detected which represent destruxins in any of the five replicate larval extractions (See supplementary figure S2) and re-analysing the data for the m/z of specific destruxins also confirmed their absence (data not shown). The LCMS system was tested using a 10fmole cytochrome C digest and 1 pmole destruxin A, however system test data was not routinely recorded and therefore the larval extracts were also analysed by MALDI ToF analysis alongside a destruxin A standard (Fig. 4). As can be seen the standard destruxin provides an excellent signal at the 1pmole level whilst the control experiment and larval extracts contain only those ions formed due to the MALDI matrix itself and no destruxin signals can be determined. This allows us to confirm the absence of destruxins, at least up to the 1pmole level per larval experiment.

### *Metarhizium* pathogenicity genes expressed in mosquito gut and faeces

**Figure 5:**
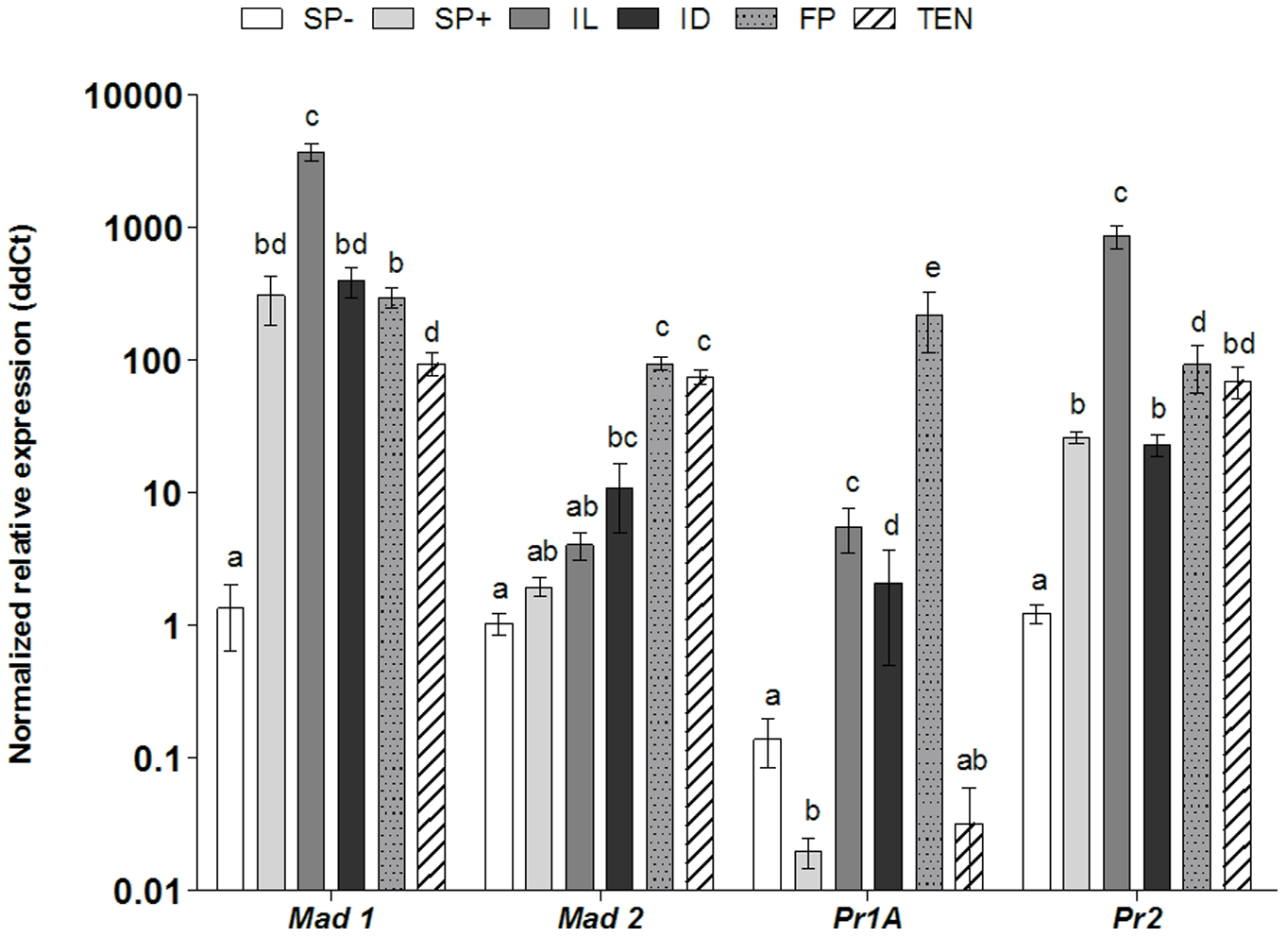
*Metarhizium* pathogenicity genes expressed in mosquito gut and faeces. Expression of protease (*Pr1A, Pr2*) and adhesin (*Mad1, Mad2*) pathogenicity related genes by conidia of *M. anisopliae*, 48 hr pi, analysed by quantitative PCR. SP−: Spore pellet in the absence of *Ae. aegypti* larvae, SP+: Spore pellet in presence of larvae, IL: infected live larvae, ID: infected dead larvae, FP: mosquito fecal pellet, TEN: *Tenebrio molitor* (terrestrial host) positive control. Data was presented as mean (± SEM) means with different letters denoting statistical differences (two-way ANOVA). Data normalized to average dCt of SP−.

Proteases (*Pr1, Pr2*) and adhesins (*Mad1, Mad2*) play a key role in fungal pathogenicity, expression of the genes for these components was analyzed and was shown to be generally much higher in the gut lumen and remained high in the fecal pellets (Fig. 5). Expression of *Pr1* was significantly lower in conidia in the presence of *Ae. aegypti* larvae compared with all the other treatments (*F*(5,47)  = 96.09, *p<*0.01) but was high in infected living, infected dead and fecal pellet samples (*F*(5,47)  = 96.09, *p<*0.05) even when compared with the *T. molitor* treatment (*F*(5,47)  = 96.09, *p<*0.001). *Pr1* expression was greatest in fecal pellets (*F*(5,47)  = 96.09, *p<*0.001) then infected larvae (*F*(5,47)  = 96.09, *p<*0.05) followed by infected dead larvae (Fig. 5). All treatments had a significantly higher expression of *Pr2* than the spore pellets not exposed to larvae with expression being highest in infected living larvae (*F*(5,47)  = 96.09, *p<*0.001) then the fecal pellet (*F*(5,47)  = 96.09, *p<*0.01; Fig. 5). Spore pellets exposed to larvae and infected dead larvae had lower and significantly similar expression levels. Conidia exposed to *T. molitor* had similar *Pr2* expression to the spore pellet exposed to the larvae, infected dead larvae and the fecal pellets (Fig. 5).

The pattern of expression of *Mad1* was comparable with the expression of *Pr2* with the exception of a slightly lower relative expression in the fecal pellet (Fig. 5). Infected living larvae showed significantly greater expression than any other treatment (*F*(5,47)  = 96.09, *p<*0.01; Fig. 5). Greatest expression levels of *Mad2* were detected in fecal pellets (*F*(5,47)  = 96.09, *p<*0.001), *T. molitor* (*F*(5,47)  = 96.09, *p<*0.001) and infected dead (*F*(5,47)  = 96.09, *p<*0.01) when compared with the spores not exposed to larvae (Fig. 5).

### Mortality linked to fungal protease-induced apoptosis

One possible mechanism that may eventually lead to mortality of the larvae is the activation of apoptotic pathways in the larvae (involving caspase enzymes) by active agents released by the conidia. As the active agents identified were proteases, the effect of inhibiting these enzymes on larval mortality was investigated and the larval caspase activity also monitored.

**Figure 6:**
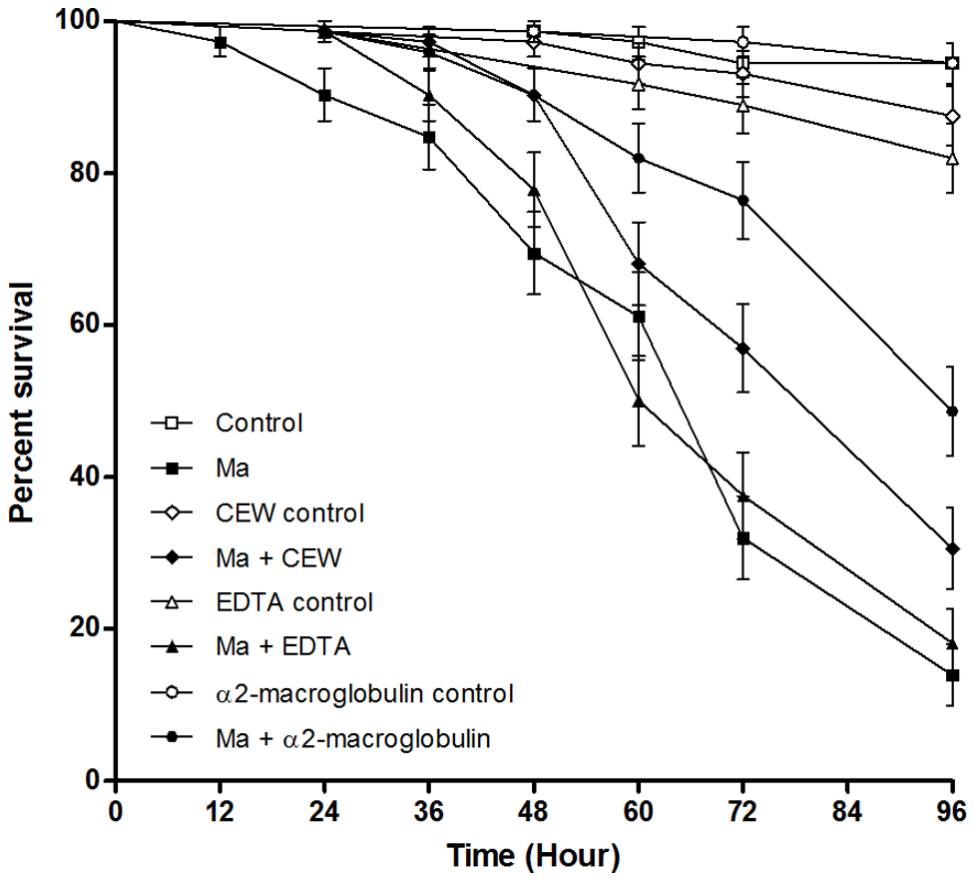
Survival of *Ae. aegypti* larvae in presence of protease inhibitor. *Ae. aegypti* larvae (n = 72) were inoculated by *M. anisopliae* with and without protease inhibitors. CEW: Chicken Egg White a *Pr1* specific inhibitor, α2 mac: α2 macroglobulin a global protease inhibitor and EDTA a metalloprotease inhibitor. Kaplan-Meier method was used to plot cumulative survival curves of larvae after inoculation, log-rank test was used to assess differences in survival between treatments. Uninhibited conidia caused greater mortality than conidia treated with inhibitors with the exception of EDTA (p<0.001). Controls consist of either 0.05% Aqueous Tween only, or 0.05% Aqueous Tween with protease inhibitor.Caspase activity, particularly caspases 3/7 and 8, was consistently higher in *Ae. aegypti* larvae exposed to live conidia compared to the heat killed conidia 48–72 hr pi (*F*(5,54)  = 203.60, *F*(5,54)  = 71.15 (caspase 3/7 and 8 respectively) *p*<0.001; Fig. 7B–D). Caspase activity elicited by heat killed conidia increased over time up to 72 hr pi.

**Figure 7:**
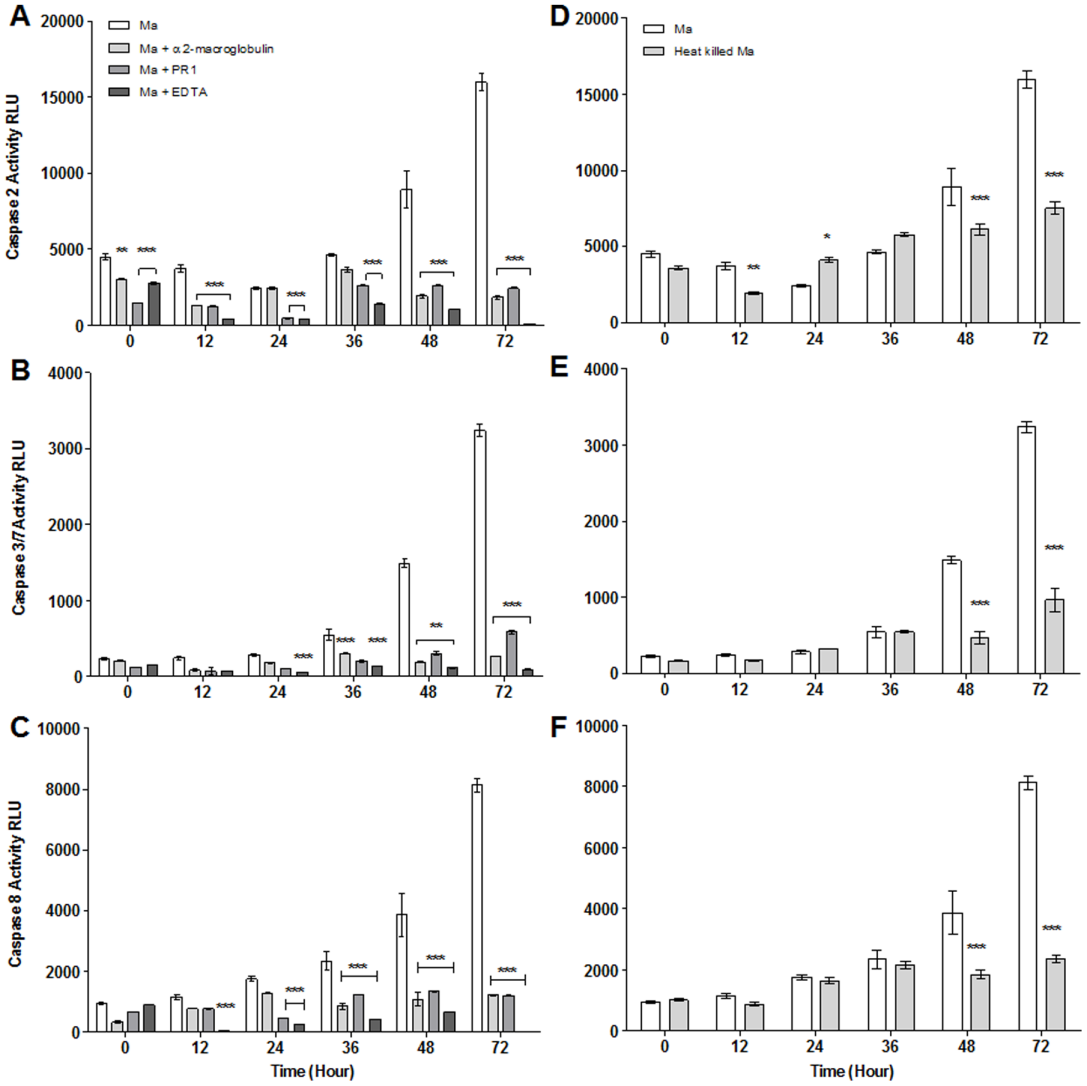
Caspase activity in *Ae. aegypti* exposed to *M. anisopliae* and protease inhibitors. Caspase activity in response to *M. anisopliae* with and without protease inhibitors (A–C) and exposed to live and heat killed conidia (D–F). Data was presented as mean (± SEM) (two-way ANOVA, ***- p<0.001, **-p<0.01, *-p<0.05, compared with *M. anisopliae* uninhibited control).

Mortality of larvae incubated with the fungus was significantly lower in the presence of protease inhibitors with the exception of EDTA which was not significantly different from the *M. anisopliae* treated larvae (Fig. 6) The inhibition with chicken egg white increased percentage survival after treatment with the fungus from approximately 10% to 30%, whilst inhibition by α2-macroglobulin improved this value to 50% of the untreated larvae. As well as the effect of the proteases produced by the fungus on larval survival, the study of the effect of the inhibition of such bioactive entities on apoptosis was studied. Activity of caspases 2, 3/7 and 8 was significantly higher in *Ae. aegypti* larvae inoculated with live conidia of *M. anisopliae* without protease inhibitors than with inhibitors (Fig. 7). Interestingly, in the *M. anisopliae* treated larvae, activity increased dramatically, concomitant with larval mortality, between 36 and 72 hr pi (Figs. 6, 7). Caspase activity was significantly lower in larvae in the presence of protease inhibitors for the whole period of the assay (*F*(5,72)  = 661.39, *F*(5,72)  = 90.4, *F*(5,72)  = 75.42 (caspase 3/7, 2 and 8 respectively) *p<*0.001; Fig. 7A, B and C). Caspase 2, 3/7 and 8 activity was generally lower 24–72 hr pi in the presence of EDTA than the other inhibitors (*F*(5,72)  = 1359.03, *F*(5,72)  = 486.01, *F*(5,72)  = 271.46 (caspase 3/7, 2 and 8 respectively) *p<*0.001; Fig. 7A, B and C).

### Oxidative stress is not an obvious mediator of apoptosis

Oxidative stress within organisms can also be a trigger for the initiation of apoptosis and therefore various indicators of such stress were studied in larvae with and without conidia. Examination of reactive oxygen species generation, lipid peroxidation, catalase, superoxide dismutase and glutathione-S-transferase activity during two days of pathogenesis revealed no major differences between uninfected and *M. anisopliae* infected mosquito larvae except glutathione-S-transferase activity which was higher (*F*(2,48)  = 12.10, *p*<0.01) 48 hr pi in infected (Figs. S1A–E). At 72 hr pi when most larvae were dead or dying we found the decrease in ROS generation (*F*(2,60) = 5.05, *p*<0.01); lipid peroxidation (*F*(2,59)  = 1.66, *p*<0.05); catalase (*F*(2,48)  = 6.77, *p*<0.01); and glutathione-S-transferase (*F*(2,48)  = 12.10, *p*<0.01) activity in infected insects (Figs. S1A−E).

### Immune and stress management systems fail to protect mosquito larvae

**Figure 8:**
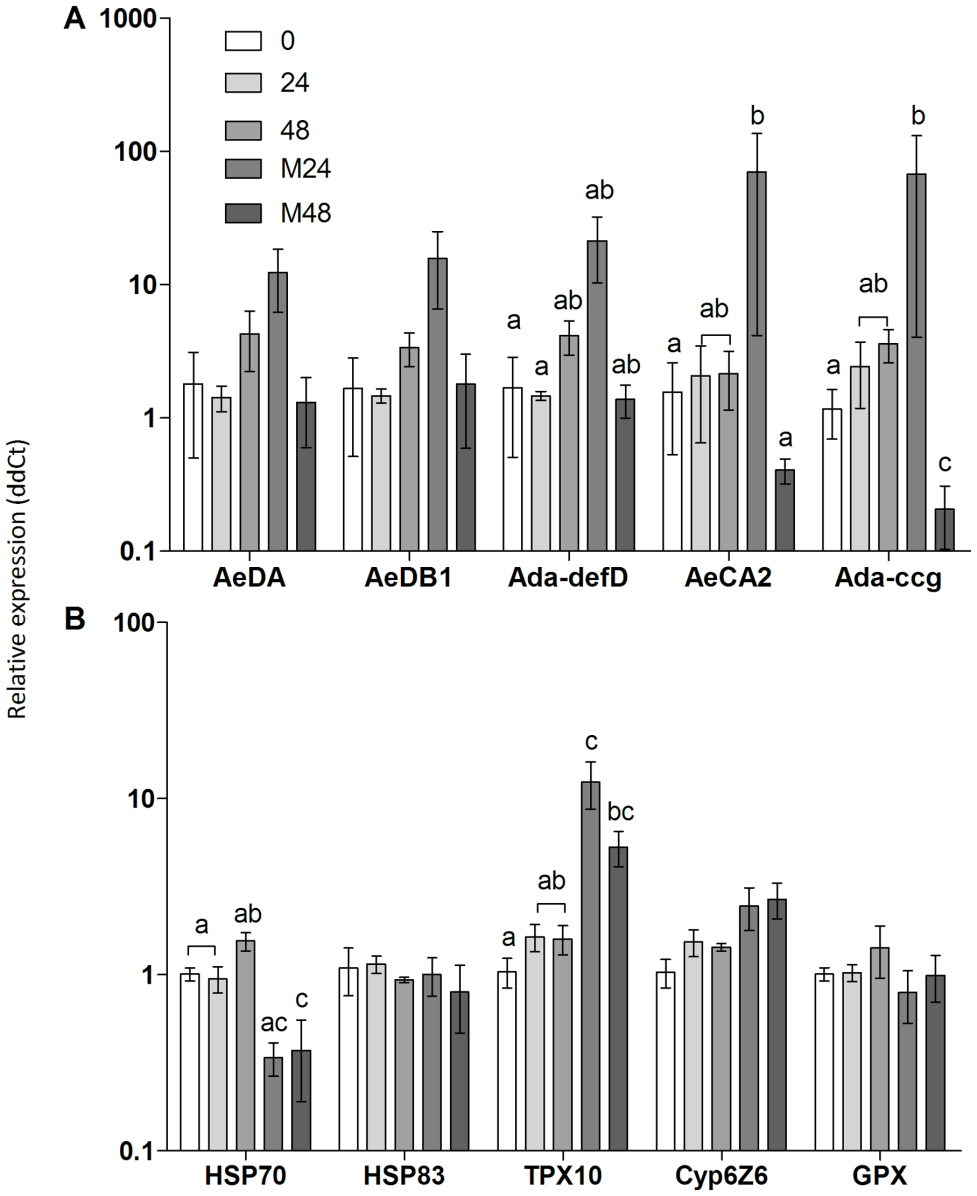
Expression of *Ae. aegypti* antimicrobial peptide and stress management genes during exposure to *M. anisopliae.* Expression of antimicrobial and stress management genes on *Ae. aegypti* were analysed in larvae inoculated with *M. anisopliae* 0, 24 and 48 hr pi by quantitative real time PCR. Antimicrobial genes included; *AeDA* (Defensin A), *AeDB1* (Defensin B), *Ada-defD* (Defensin D), *AeCA2* (Cecropin A), *Ada-ccg* (Cecropin G) and stress management genes; *HSP70* (Heatshock protein 70), *HSP83* (Heatshock protein 83), *TPX10* (Thiol peroxidase 10), *Cyp6Z6* (Cytochrome P450), *GPX* (Glutathione peroxidase). Data was presented as mean (± SEM) means with different letters are statistically different (two-way ANOVA)

Given the release of active proteases by the fungus and elicitation of a pathogenic response when exposed to the larvae, the study of the larval response to the fungus was also undertaken. The analysis examined the larval defense mechanisms (predominantly antimicrobial peptides) as well as the stress response of the larvae. Expression of the antimicrobial peptide (AMP) genes *AeDA* and *AeDB* was not significantly up regulated following *Ae. aegypti* larval ingestion of live *M. anisopliae* conidia 24 hr pi (Fig. 8A). While expression levels of *Ada-DefD* and *Ada-CcG* was significantly greater in samples exposed to *M. anisopliae* after 24 hr compared with time zero (*F*(4,50)  = 16.12, *p*<0.05) it was not significantly different from the 24 hr untreated control (Fig. 8A). However, the gene was significantly down regulated in larvae exposed to *M. anisopliae* 48 hr pi compared with the other treatments (*F*(4,50)  = 16.12, *p*<0.05; Fig. 8A). *AeCA2* was significantly down regulated in larvae at 48 hr pi compared with 24 hr pi (*F*(4,50)  = 16.12, *p*<0.01) but these did not differ from unexposed larvae at 48 hr and 24 hr, respectively (Fig. 8A).

Of the five stress management genes examined, a significant increase in expression of the *TPX10* was observed in larvae exposed to *M. anisopliae* 24 hr compared with the time zero (*F*(4,40)  = 7.71, *p*<0.001) and 24 hr untreated control (*F*(4,40)  = 7.71, *p*<0.01; Fig. 8B). While there was a significant increase in expression of *TPX10* at 48 hr in exposed larvae compared with time zero (*F*(4,40)  = 7.71, *p*<0.05) this was not significant compared with the unexposed larvae at 48 hr (Fig. 8B). In contrast, the *Hsp70* gene was down-regulated in larvae exposed to conidia 48 hr pi compared with the untreated control (*F*(4,40)  = 7.71, *p*<0.01; Fig. 8B). No significant changes were observed for both AMPs and stress genes in the untreated controls at 0, 24 and 48 hr (Fig. 8A and B).

## Discussion

This study shows that conidia of *M. anisopliae* do not firmly adhere to the surface of the cuticle of *Ae. aegypti* larvae and do not gain entry by penetrating the host cuticle. Conidia have been reported adhering to the cuticle, particularly the siphon and mouthparts of the fungus [21], thereby infecting the larvae in a similar manner with which it infects terrestrial hosts [2].

Conidia attach to terrestrial hosts initially *via* passive hydrophobic forces followed by secretion of enzymes and adhesion compounds to anchor the spore to the cuticle surface [6], [7]. The failure of conidia to adhere to the cuticle of terrestrial hosts has been attributed to the cuticle chemistry, with some compounds altering hydrophobicity or being fungistatic [6], [22]. It is feasible that the mosquito larval cuticle is not conducive for adhesion, with any mucilage produced by the fungus being diluted in the water. In contrast, aquatic pathogens of mosquitoes such as *Lagenidium giganteum* (Oomycetes) and *Coelomomyces punctatus* (Chytridiomycetes) produce zoospores that can attach to and penetrate the larval cuticle before colonizing the hemocoel. *Culicinomyces clavisporus*, an aquatic Sordariomycete related to *M. anisopliae*, produces conidia which, following ingestion by the larvae, adhere to and penetrate through the chitinous wall in the fore- and hindgut [23].

In conidia of *M. anisopliae, Mad1* expression in the presence of mosquito larvae suggest the fungus had responded to cuticular cues in a similar manner to a terrestrial host despite its failure to adhere through passive hydrophobic forces. *Mad1* was up regulated particularly inside the gut of live insects suggesting that the fungus had perceived additional cues. *Mad2* was not upregulated in the same manner, however, expression of both these genes was significantly higher in the gut of dead insects and fecal pellets possibly due to nutritional stress which would also explain why no germ tubes were produced. Nutrient starvation is known to up regulate *Mad2* [9]. The concomitant upregulation of *Mad1, Mad2, Pr1* and *Pr2* by the ungerminated conidia of *M. anisopliae* suggest that the fungus is mounting a response to infect but fails to progress due to the lack of stimuli normally present in the terrestrial arthropod host. Conidia of *M. anisopliae* are readily ingested by mosquito larvae with some workers suggesting this to be the main route of infection [3]. Our studies show that the conidia failed to produce germ tubes and penetrate the gut wall, nor do they cause inflammation of the midgut epithelium or interfere with gut function, allowing the insect to remove conidia in compact fecal pellets at least until death. Toxins, particularly destruxins, have been implicated as the cause of mosquito larval death, produced by ungerminated conidia on the cuticle, inside the gut or released following digestion of *M. anisopliae* conidia [13], [24], [25]. In our study, no destruxins were detected in *Ae. aegypti* larvae that had ingested conidia of *M. anisopliae* 4556, even though this strain is known to produce destruxins, thus discounting these compounds as the cause of death.

Extracellular proteases of *M. anisopliae,* with the exception of metalloproteases, contribute significantly to *Ae. aegypti* larval mortality which appears to be mediated through stress induced apoptosis. *Pr1* and *Pr2* were expressed during passage through the insect gut, in the fecal pellet and recently killed larvae. Chicken egg white, an inhibitor of *Pr1*, significantly improved survival of *Ae. aegypti* larvae but not to the same extent as the global protease inhibitor, α2 macroglobulin, suggesting that several proteases working in concert were contributing to larval mortality. Not all proteases contribute to mortality since inhibition of *Pr2* did not improve survival (unpublished). EDTA treated insects posed an anomaly as these exhibited low caspase activity but high larval mortality. It is possible that EDTA, besides inhibiting metalloproteases, interfered with cation dependent cellular processes such as signalling, homeostasis, and caspase activation [25] which would exacerbate the stress caused by the fungal pathogen. This is clearly an area for further investigation. Mortality in heat killed conidia and untreated control was statistically similar suggesting that extracellular proteases contributed significantly to larval mortality. Proteases will accrue with time as more conidia pass through the gut. The high survival of larvae in the presence of protease inhibitors and heat killed conidia show that death does not arise due to blockage of the mouthparts or breathing apparatus as suggested by some previous studies [11].

Upregulation of *A. aegypti* antimicrobial peptide (AMP) genes, peaking 24 hr pi, is the typical immune response of insects exposed to pathogens, stress or injury [18]. The mosquito larvae did not mount a strong AMP mediated defense response to *M. anisopliae;* the only significant activity was downregulation of cecropins A and G, 48 hr pi, which coincided with a significant increase in caspase activity and larval mortality. Indeed, mortality appeared to be correlated with caspase activity. Activities of initiator (caspases 2 and 8) and effector (caspases 3 and 7) caspases increased with time suggesting an increasing number of cells undergoing apoptosis. Once a threshold of dead cells had been reached the insect would be unable to sustain life functions resulting in death. Apoptosis is known to be induced by oxidative damage either from oxygen free radicals or hydrogen peroxide directly or from their generation in cells by injurious agents [26]. Insects, like many other organisms, actively produce reactive oxygen intermediates as signalling molecules to control processes such as, apoptosis, abiotic stress responses, and pathogen defense [18], [26]. Cellular antioxidant mechanisms countering oxidative stress include soluble free radical scavenger molecules such as glutathione and enzymes like superoxide dismutases, catalases and peroxidases. Most of these enzymes were not elevated in *M. anisopliae* infected *Ae. aegypti* larvae, with the exception of glutathione-S-transferase, 48 hr pi but at 72 hr pi they had all fallen possibly due to insects being close to death. Expression of the stress management genes at the critical 48 hr pi was not as extensive as reported in terrestrial insects [26], presumably due to the mosquito larvae never encountering the fungus and evolving an appropriate response. Most notable was the downregulation of *Hsp70* and upregulation of *TPX10*. *Hsp70* has vital housekeeping functions, maintaining homeostasis and protecting cells against thermal and oxidative stress [27]. It can directly inhibit apoptosis upstream of caspase 3 activation [27], [28]. *Hsp70* is activated by a wide range of factors including cytokines, energy (ATP) depletion and reactive oxygen species [27]. The downregulation of *Hsp70* would predispose the mosquito larvae to apoptosis. Thiol peroxidases (TPx) play an important antioxidant role in a wide range of organisms including insects. They utilize thioredoxin as a substrate to carry out detoxification of reactive oxygen species [29]. Thiol peroxidases can inhibit apoptosis [29], therefore, upregulation of *TPX10* may be an attempt by the *M. anisopliae* infected larvae to contain apoptosis.

This study shows for the first time that mortality of mosquito larvae exposed to *M. anisopliae* is multifactorial. It is not due to invasion and colonisation of the host, as proposed by other workers, but entails *M. anisopliae* proteases triggering stress induced apoptosis which ultimately leads to host death, hence the verdict of accidental death. The fungus has the machinery to infect terrestrial insect hosts and although some of this apparatus is expressed in the mosquito larvae it is ineffective in the aquatic environment. Likewise, the mosquito larvae did not mount a strong defenseresponse as for *C. clavisporus* [23]. Presumably, mosquito larvae have either not evolved appropriate pathogen recognition receptors to identify *M. anisopliae* derived pathogenicity associated molecular patterns, as is the case for terrestrial hosts [30] or alternatively, the lack of success with regard to the fungal colonization limits the insects ability to recognise the attempted infection. Failure of *M. anisopliae* to colonize and sporulate on the mosquito host would result in no horizontal transfer of inoculum and for biocontrol management strategies would require regular application unlike the aquatic pathogens which can cause epizootics because of their ability to reproduce in mosquitoes and other aquatic invertebrates [3]. Genetic or physiological manipulation of *M. anisopliae* to over produce proteases could accelerate larval mortality and pose little environmental risks because of the inability of the fungus to infect or reproduce in mosquito larvae.

## Supporting Information

File S1 **Figure S1-S2, Table S1, Text S1-S5. Figure S1.** Limited antioxidant activity in mosquito larvae exposed to *M. anisopliae*. Activity of mosquito larvae exposed and not exposed to conidia of *M. anisopliae*. (A) Reactive oxygen species (ROS) generation, and activity of (B) MDA (lipid peroxidation), (C) catalase, (D) Superoxide dismutase (SOD),and (E) glutathione-S-transferase (GST). Data presented as mean ± (SEM) (Two-way ANOVA, **-p<0.01, *-p<0.05 compared with uninfected for the same time point). **Figure S2.** LCMS chromatogram showing no detectable *Metarhizium anisopliae* destruxin in *Aedes aegypti* larval extracts. **Table S1.** *Metarhizium anisopliae* and *Aedes aegypti* loci used for expression analysis. **Text S1.** Cryo-SEM. **Text S2.** Analysis of destruxins. **Text S3.** Enzyme and enzyme inhibitor assays. **Text S4.** Transcript quantification of insect and fungus-derived genes. **Text S5.** ROS production, lipid peroxidation and antioxidant system activity.
(DOCX)
