# Supplementary material for: Metarhizium anisopliae Pathogenesis of Mosquito Larvae: A Verdict of Accidental Death
Source: PLoS One. 2013 Dec 13;8(12):e81686. doi: 10.1371/journal.pone.0081686 (PMC3862491; doi:10.1371/journal.pone.0081686)
Supplement: File S1 — Figure S1-S2, Table S1, Text S1-S5. Figure S1. Limited antioxidant activity in mosquito larvae exposed to M. anisopliae. Activity of mosquito larvae exposed and not exposed to conidia of M. anisopliae. (A) Reactive oxygen species (ROS) generation, and activity of (B) MDA (lipid peroxidation), (C) catalase, (D) Superoxide dismutase (SOD),and (E) glutathione-S-transferase (GST). Data presented as mean ± (SEM) (Two-way ANOVA, **-p<0.01, *-p<0.05 compared with uninfected for the same time point). Figure S2. LCMS chromatogram showing no detectable Metarhizium anisopliae destruxin in Aedes aegypti larval extracts. Table S1. Metarhizium anisopliae and Aedes aegypti loci used for expression analysis. Text S1. Cryo-SEM. Text S2. Analysis of destruxins. Text S3. Enzyme and enzyme inhibitor assays. Text S4. Transcript quantification of insect and fungus-derived genes. Text S5. ROS production, lipid peroxidation and antioxidant system activity. (DOCX) [file pone.0081686.s001.docx]

**SUPPORTING INFORMATION TEXT**

##### Experimental Procedures

**Text S1 Cryo-SEM**

The larvae were mounted on SEM sample holders using cryogenic glue, both horizontally for imaging of the cuticle and vertically for fracturing and cross sectional imaging before being plunged into a nitrogen slush for rapid freezing then transferred to the cryogenic preparation chamber in vacuum. The specimens were warmed up to -90°C for 10 minutes to remove surface ice then returned to -130°C. Fracturing of the vertically mounted larvae was performed inside the preparation chamber at -130°C using a rotating knife. The specimens were then coated with approximately 5nm of Platinum then transferred to the SEM stage. Imaging took place at -130°C.

**Text S2. Analysis of destruxins**

Aedes aegypti larvae that had been exposed to M. anisopliae were washed three times in distilled water to remove spores adhering to the cuticle surface, then ten larvae from each replicate were placed in separate Eppendorf tubes and homogenised with a micropestle. Eppendorf tubes were charged with 0.5 ml dichloromethane (CH_2_Cl_2_)/ethyl acetate (EtOAc) (1:1, v/v) and agitated for 5 minutes before leaving to rest for 30 minutes. After 30 minutes the supernatant was removed and discarded and another 0.5 ml CH_2_Cl_2_/EtOAc was added to the sample and the step before was repeated. Once the second supernatant was removed the remaining solution was evaporated using a speed vacuum. Extracts were stored at -20°C until required for analysis. Destruxin extracts were analyzed by nano-reverse phase liquid chromatography (Ultimate Pump, LC-Packing, Dionex, The Netherlands) using an electrospray ion trap MS (LCQ Deca XP, ThermoElectron, Hemel Hempstead, UK). LC-ESI MS/MS separations were performed using a 10cm x 75mm I.D. pulled-tip capillary column, that was slurry packed in-house with 3µm, 300Å pore size C18 silica bonded stationary phase (PepMap, Dionex, Camberley, UK). The autosampler was fitted with a 5 µL injection loop and was refrigerated at 4ºC during analysis. After injecting 5 µL of extract, the analytes were eluted from the analytical column, over which a 15min wash with 98% buffer A (0.1% formic acid in water v/v) was applied and any destruxins present were then eluted using a stepwise gradient of 0% solvent B (0.1% formic acid in acetonitrile v/v) to 65% solvent B in 100min and then to 100% B in 10min with a constant flow rate of 0.2 µL/min. The electrospray MS was operated in a data-dependent mode in which each full MS scan (m/z 400-1500) was followed by three MS/MS scans, in which the three most abundant peptide molecular ions were dynamically selected for collision-induced dissociation (CID) using a normalized collision energy of 35%. The temperature of the heated capillary and electrospray voltage was 160ºC and 1.6kV, respectively. Post-acquisition processing utilised previously reported diagnostic mass losses during MS/MS analysis in order to select for destruxins from the mixture of separated ions (Butt et al., 2009) and the sensitivity of the instrument was tested with a 10fmole/injection sample of cytochrome C tryptic digest and 1pmole/injection destruxin A standard before analysis (Sigma, UK).

**Text S3. Enzyme and enzyme inhibitor assays**

**(i) *Pr1* protease activity.** Pr1 bound to conidia and secreted in the water bathing the larvae was assessed at 0, 48 and 72 hr pi using methods adapted from Shah *et al*., (2005). Buffered substrate was used as a control.

**(ii) Protease inhibition assays.** *Ae. aegypti* larvae were exposed to conidia (1x10^7^ conidia ml^-1^) of *M. anisopliae* ARSEF 4556 suspended in 1 ml of buffer (0.03% aqueous Tween 80) containing either chicken egg white (Pr1) inhibitor (0.1 mg/ml), EDTA (1 mM) or α2-macroglobulin inhibitor (1 µg/ml) which were specific for *Pr1*, metalloprotease and global (serine, cysteine, metallo-) proteases, respectively. All the inhibitors were purchased from Sigma-Aldrich. Controls consisted of buffer and buffered inhibitor. Mortality was recorded at 0, 12, 24, 36, 48 and 72 hr pi. Assays were also done using heat killed conidia at 10^7^ conidia ml^-1^.

**(iii) Caspase activity.** This was assessed in six *Ae. aegypti* larvae per treatment. Larvae were incubated in a spore suspension of *M. anisopliae* at room temperature and at 0, 12, 24, 36, 48 and 72 hr pi removed and rapidly frozen in liquid nitrogen. Larvae were homogenized mechanically using a standard elution volume cuvette and plunger (Promega). Total homogenates were resuspended in 420 µL 0.5% Triton lysis buffer (Tris 20mM, NaCl 100mM, EDTA 500mM, 0.5% Triton X-100), agitated gently before incubating on ice for 10 min. Homogenates were then centrifuged at 14, 000 g for 10 min and 35 µL aliquots of supernatant were added to 4 replicate wells in a white walled 96-well microtiter plate (Costar, Corning). Luminometric assays for caspase-2, caspase-3, caspase-7 and caspase-8 activity were performed in accordance with the manufacturer’s guidelines using the Caspase Glo 2, Caspase Glo 3-7 and Caspase Glo 8 assay kits (Promega) by adding 35 µL of Caspase Glo reagent to the sample. Plates were agitated gently for 30 s before incubating at room temperature. Endpoint luminescence was measured after 1 hr.

**Text S4 Transcript quantification of insect and fungus-derived genes**

**(i) *Samples, RNA extraction and cDNAsynthesis:*** *Ae. aegypti* larvae (L_3-4_) were exposed to *M. anisopliae* ARSEF 4556 (10^7^ conidia ml^-1^), controls not exposed to fungus were also included. For the transcript analysis of insect-derived genes, ten larvae were placed in 10 ml distilled water in 6-well Nunc cell culture multidishes (128 x 86 cm, Thermo Scientific) and incubated for 48 hr at room temperature. Six replicates were harvested immediately before treatment (time 0) and after 24 and 48 hr, dead larvae were removed and the remaining live insects were pooled and divided into batched of 10 per sample and frozen under liquid nitrogen and stored at -80°C until required.

For the transcript analysis of fungus-derived genes, ten larvae were placed into 100 ml distilled water in 250 ml circular plastic containers with a perforated lid and incubated for 48 hr at room temperature, *Ae. aegypti* larvae exposed to *M. anisopliae* ARSEF 4556 were divided into living and dead samples. Other samples included: *Ae. aegypti* larvae not exposed to fungus, *M. anisopliae* ARSEF 4556 conidia added in the absence of larvae, fecal pellets, and a terrestrial host (*Tenebrio molitor*) positive control. Each sample was frozen under liquid nitrogen, freeze dried and stored at -80°C until required; three replicate treatments were made for each sample.

Fecal pellets were harvested from larvae previously starved for 48 hr and inoculated with *M. anisopliae* ARSEF 4556 conidia. Pellets were harvested 24 hr pi and washed three times in distilled water and centrifuged to collect. *T. molitor* adults were immersed in 50 ml water containing 10^7^ conidia ml^-1^ for 20 sec and incubated on moist filter paper at 27°C for 48 hr.

Samples were ground with a micropestle and total RNA extractions carried out using the RNeasy Micro kit (Qiagen) following manufacturer’s instructions. An RNA carrier was also used for samples from the experiment to measure *Ae. aegypti*-derived genes according to manufacturer’s instructions (Qiagen). RNA concentration and purity was assessed at 260 and 280 nm absorbance using a Nanophotometer (Implen).

**(ii) Quantitative PCR (qPCR):** Optimal primer concentration and annealing temperature was determined using samples expected to contain the genes of interest. qPCRs were performed in 10 μl reactions (4 μl diluted cDNA, 1 μl optimised concentration primers and 5 μl Roto-Gene SYBR Green PCR mix (Qiagen) with the Rotor-Gene 6000, and 3 μl cDNA diluted cDNA, 1 μM primers and 5 μl SYBR Green Fast mix (Quanta) for the CFX96™ system).

For *Ae. aegypti* genes *ada-rb*7, *ada-rp*49, *ae-def*A, *ae-def*B, *ada-ccg*, *ae-ca2,* *hsp*70, *hsp*83, *cyp*6z6, *tpx* and *gpx* cycling conditions 95°C 5 minutes, 42 cycles 95°C 5 sec, primer annealing (65-59°C)10 sec, 72°C 20 sec were used. A touchdown profile was used for *ada-def*D and *aec*A2 where the annealing temperature was reduced from 65°C by 1°C per cycle for the first 5 cycles. For *M. anisopliae* genes *18S rRNA*, *tef*, *Pr1*A, *Pr2*, *Mad1* and *Mad2* cycling conditions 45°C 5 min, 95°C 3 min, and 39 cycles of 95°C 10 sec, 60.3°C 10 sec and 72°C 30 sec was used. A high resolution melt curve (HRM) analysis was included to all reactions to ensure no spurious products were present. A non-template water negative control and a two-fold dilution series of pooled cDNA samples to provide a standard curve for each primer set was also included, each replicate sample was ran in duplicate..

**Text S5 ROS production, lipid peroxidation and antioxidant system activity**

The 2´,7´-dichlorodihydroﬂuorescein (DCFH_2_) was used for in vitro assay for measuring total ROS/RNS free radical activity in the homogenates (Chen *et al*., 2010). Five µL of samples were added to the wells with 200 µL of the DCFH_2_ solution (10 µM in PBS) and the oxidation reaction is allowed to proceed 30 min 37°C. Samples were measured fluorometrically (Ex/Em = 485/530 nm). The excitation ﬁlter was set at 485 nm and the emission ﬁlter was set at 530 nm. The ROS generation is presented as ﬂuorescence ((Ft_30_ - Ft_0_), where Ft_30_ = ﬂuorescence at time 30 min and Ft_0_ = ﬂuorescence at time 0 min) per mg protein. Superoxide dismutase (SOD) activity was determined as the suppression of the reduction rate of NBT by the superoxide anion, generated as a result of xanthine oxidation by xanthine oxidase (McCord & Fridovich, 1969). 10 µL of the sample was mixed with 150 µL of the reacting solution (70 µM of NBT; 125 µM of xanthine; both dissolved in PBS) and 2 µL xanthine oxidase solution (10 mg of albumin bovine (BSA); 100 µL of xanthine oxidase (4.11 unitmL^-1^); dissolved in 1 mL of PBS). The mixture was incubated in darkness at 28°С for 60 min. SOD activity is presented as the difference in absorbance between a sample containing the mixture and a clean reagent mixture at ΔA 560nm /min/mg protein. Catalase activity was estimated as the decomposition rate of hydrogen peroxide (Wang *et al*., 2001). 500 µL of substrate (1% hydrogen peroxide in PBS) was mixed with 1 µL of homogenate. The mixture was incubated at 28°С for 60 min. The catalase activity is presented as the ΔA at 240nm/min /mg protein. Glutathione-S-transferase (GST) activity was determined as the changing of the concentration of 5-(2,4-dinitrophenil)-glutathione (product of DNCB and glutathione interaction) catalyzed by GST (Habig *et al*., 1974). The reaction mixture contained 1mM glutathione and 1mM of DNCB and 1 µL of the sample in 200 µL PBS. The mixture was incubated 30 min at 28°С. GST activity is represented as ΔA at 340nm/min /mg protein. The process of lipid peroxidation results in the formation of MDA. This is a later product in the sequence of lipid peroxidation reactions (Rael *et al*., 2004). The TBA assay was used to assess the MDA concentration, with some modifications as described in Dubovskiy *et al*. (2008). 20 µL of 20 % trichloroacetic acid was mixed with 40 µL of the sample, after which the mixture was centrifuged at 10000 g for 10 min at +4^o^C. 50 µL of supernatant was mixed with 150 µL of 0.8 % TBA, and the mixture was incubated at +100°C for 60 min. The MDA-TBA adduct was quantified fluorometrically (Ex/Em = 532/553 nm). The MDA concentration is presented as nmoles of MDA per mg protein using the 1,1,3,3-tetramethoxypropane as a standard. The concentration of protein in the homogenates was determined by the Bradford method (Bradford, 1976); BSA was used to construct the calibration curve. All assays were made with Biotek Synergy H1 plate reader.

**SUPPORTING INFORMATION REFERENCES**

1. Bonizzoni M, Dunn WA, Campbell CL, Olson KE, Dimon MT, Marinotti O, James AA (2011) RNA-seq analyses of blood-induced changes in gene expression in the mosquito vector species, *Aedes aegypti*. *BMC genomics*, **12**, 82.
2. Bradford MM (1976) “A rapid and sensitive method for the quantitation of microgram quantities of protein utilizing the principle of protein-dye binding.” *Anal. Biochem.* 72, 248-254
3. Butt TM, Ben N, Hadj E, Skrobek A, Ravensberg WJ, Wang C, Lange, CM, Vey A, Shah U-K, Dudley E (2009) Mass spectrometry as a tool for the selective profiling of destruxins ; their first identification in *Lecanicillium longisporum*. *Rapid Communications in Mass Spectrometry*, 1426-1434.
4. Chen X, Zhong Z, Xu Z, Chen L, Wang Y (2010) 2′,7′-Dichlorodihydrofluorescein as a fluorescent probe for reactive oxygen species measurement: Forty years of application and controversy. *Free Radical Research,* V. 44, 587-604.
5. **Dubovskiy IM,** Martemyanov BB, Vorontsova YL, Rantala MJ, Gryzanova EV, & Glupov VV, (**2008)** Effect of the bacterial infection on the antioxidant activity and lipid peroxidation in the midgut of larvae *Galleria mellonella* L. (Lepidoptera, Pyralidae). *Comparative Biochemistry and Physiology* Part C 148, 1–5.
6. Habig WH, Pabst, MJ, Jakoby WB (1974) Glutathione-S-transferases. *J. Biol. Chem.* 249, 7130–7139.
7. McCord JM, Fridovich I (1969) Superoxide dismutase: an enzymic function for erythro-cuprein (hemocuprein). *J. Biol. Chem.* 244, 6049–6055.
8. Muturi EJ, Kim C-H, Alto BW, Berenbaum MR, Schuler MA (2011) Larval environmental stress alters *Aedes aegypti* competence for Sindbis virus. *Tropical medicine & international health : TM & IH*, **16**, 955–64.
9. Pan X, Zhou G, Wu J, Bian G, Lu P, Raikhel AS, Xi Z (2012) *Wolbachia* induces reactive oxygen species (ROS)-dependent activation of the Toll pathway to control dengue virus in the mosquito *Aedes aegypti*. *Proceedings of the National Academy of Sciences of the United States of America*, **109**, E23–31.
10. Rael LT, Thomas GW, Craun ML, Curtis CG, Bar-Or R, Bar-Or D (2004) Lipid peroxidation and the thiobarbituric acid assay: standardization of the assay when using saturated and unsaturated fatty acids. *J. Biochem. Mol. Biol*. 37, 749–752.
11. Shah F, Wang C, Butt TM (2005) Nutrition influences growth and virulence of the insect-pathogenic fungus *Metarhizium anisopliae*. *FEMS Microbiology Letters* **251**, 259–266.
12. Telang A, Qayum AA, Parker A, Sacchetta BR, Byrnes GR (2011) Larval nutritional stress affects vector immune traits in adult yellow fever mosquito *Aedes aegypti* (*Stegomyia aegypti*). *Medical and veterinary entomology*, 1–11.
13. [Wang](http://www.scopus.com/search/submit/author.url?author=Wang+Y.&origin=resultslist&authorId=8950561800) Y, Oberley LW, [Murhammer DW](http://www.scopus.com/search/submit/author.url?author=Murhammer+D.W.&origin=resultslist&authorId=7004909813) (2001) [Evidence of oxidative stress following the viral infection of two lepidopteran insect cell lines](http://www.scopus.com/record/display.url?eid=2-s2.0-0035578258&origin=resultslist&sort=plf-f&cite=2-s2.0-0035578258&src=s&imp=t&sid=98039C632128F9C08FC4DE8642392964.WXhD7YyTQ6A7Pvk9AlA%3a20&sot=cite&sdt=a&sl=0). *Free Radical Biology and Medicine*, 31, 1448-1455
14. Xi Z, Ramirez JL, Dimopoulos G (2008) The *Aedes aegypti* toll pathway controls dengue virus infection. *PLoS pathogens*, **4**, e1000098.
15. Zhao L, Becnel JJ, Clark GG, Linthicum KJ (2010) Expression of *AeaHsp26* and *AeaHsp83* in *Aedes aegypti* (Diptera: Culicidae) Larvae and Pupae in Response to Heat Shock Stress. *Journal of Medical Entomology*, **47**, 367–375.

**SUPPLEMENTAL FIGURE**

**Figure S1. Limited antioxidant activity in mosquito larvae exposed to *M. anisopliae*.**

**Figure S2** LCMS chromatogram showing no detectable *Metarhizium anisopliae* destruxin in *Aedes aegypti* larval extracts.

**
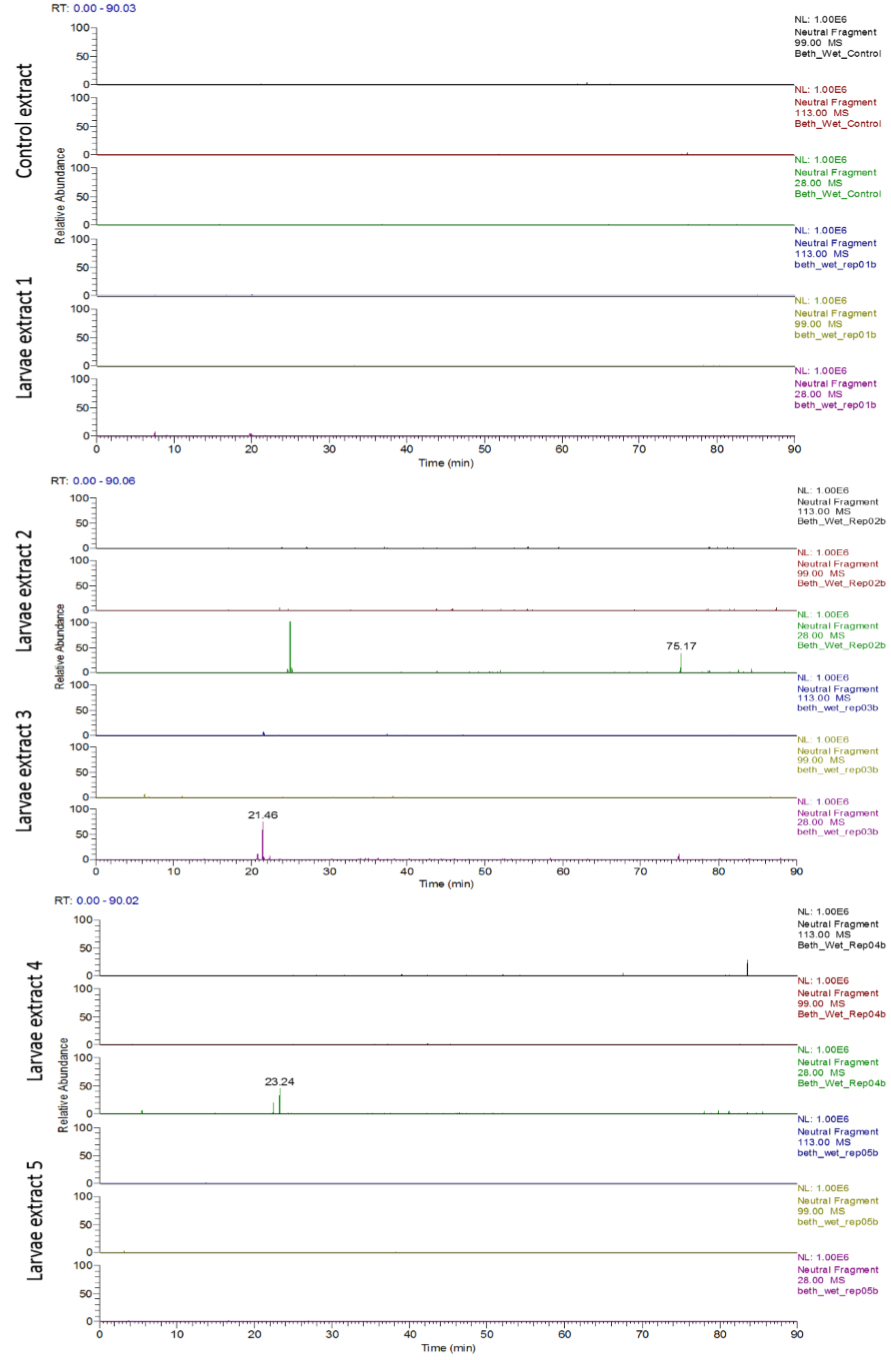
**

| Table S1. *Metarhizium anisopliae* and *Aedes aegypti* loci used for expression analysis | | | |  |  |
| --- | --- | --- | --- | --- | --- |
| **Putative function** | **Locus** | **Primer name** | **Forward primer** | **Reverse Primer** | **Sequence reference** |
| Housekeeping (*Ae. aegypti*) | Ribosomal S7 | Ada-Rb7 | TCAGTGTACAAGAAGCTGACCGGA | TTCCGCGCGCGCTCACTTATTAGATT | (Telang *et al*., 2011) |
|  | Ribosomal protein 49 | Ad_RP49 | ACAAGCTTGCCCCCAACT | CCGTAACCGATGTTTGGC | (Bonizzoni *et al*., 2011) |
|  | /L32 |  |  |  |  |
| Antimicrobial peptides (*Ae. aegypti*) | AeDA, Defensin A | AeDA | CCGAAAGGACCAACCATGAA | ATTCCGACAGACGCACACCCT |  |
|  | AeDB, Defensin B | AeDB | TCATTTGTTTCCTGGCTCTGTG | GCGGCCTGATAGGTTTCCTC |  |
|  | Ada-Defensin D | Ada-DefD | CGGTGCTGGCGGACGAA | GCAATGAGCAGCACAAGCACTATC | (Telang *et al*., 2011) |
|  | Cecropin A | AeCA2 | TGGCTGTTCTTCTCCTGA | AAAACTCGTTTTCCTGCAC |  |
|  | Cecropin G | Ada-CcG | TCACAAAGTTATTTCTCCTGATCG | GCTTTAGCCCCAGCTACAAC | (Telang *et al*. 2011), (Xi et al., 2008) |
| Stress | Hsp70, heat shock protein 70 | Hsp70 | CCCGTCCTACGTGGCGTTCA | GGTGGCCTGACGTTGCGAGT | (Muturi *et al*., 2011) |
| (*Ae. aegypti*) | Hsp83, heat shock protein 83 | Hsp83 | AAGGCCGTTAAGGATCTGGT | CGCTAGTGTGGGGAAGAGAG | (Zhao *et al*., 2010) |
| Reactive oxygen | TPX, Thiol peroxidase | TPX | TCGACCGACAGTCACTTCAC | CTGGCGGAGATTCTGCTTAC | (Pan *et al*., 2012) |
| (*Ae. aegypti*) | CYP6Z6, Cytochrome P450 | CYP6Z6 | CTGCCTTATTTGGACTTATGC | ATCACAACACTGGATTCTGG | (Muturi *et al*., 2011) |
|  | GPX, Glutathione peroxidase | GPX | ATATGGCGAAACGGAAGGTC | TCCCCGTTGACGTATATCTTG | (Pan *et al*., 2012) |
| Proteases (*M. anisopliae*) | PR1a | PR1a | TCCGAGTCCTCTTGCCTATCA | GGCACCGTTGTAGGCAAGGTAGTT |  |
|  | PR2 | PR2 | TACGCCACATTGCCAGA | GCATGTCGCACGATCAA |  |
| Adhesion genes (*M. anisopliae*) | MAD1 | MAD1 | CTCCTCACATCACCCAGGTT | GGGAGTAGGCATGACGATGT |  |
|  | MAD2 | MAD2 | CTATGTCCACCTTGCGACT | AGCAGCTGATGAGGGTCT |  |
| Housekeeping genes (*M. anisopliae*) | 18s |  | CGAAAGTCGCAATGGCTCA | CCGAAGTCGGGATTTTTAGC |  |
|  | Translation elongation factor | MaHKtEF | CGAGCGTGAGCGTGGTA | CAGCCTCGAACTCACCAG |  |
